# The Relation Between Deaf Patients and the Doctor

**DOI:** 10.1016/S1808-8694(15)30846-6

**Published:** 2015-10-18

**Authors:** Neuma Chaveiro, Celmo Celeno Porto, Maria Alves Barbosa

**Affiliations:** 1 Master’s degree student in the Health Sciences postgraduate program, Universidade Federal de Goiás. Speech therapist and LIBRAS (Brazilian sign language) interpreter, Centro Estadual de Apoio ao Deficiente.; 2 Professor Emeritus, Universidade Federal de Goiás Medical School. Coordinator of the Health Sciences postgraduate program, Universidade Federal de Goiás.; 3 Adjunct professor III, doctoral professor of the Universidade Federal de Goiás Nursing School. Teacher in the Nursing School. Universidade Federal de Goiás - Health Sciences postgraduate program. Universidade Federal de Goiás Medical School. Address: 1ª Avenida S/N - Setor Universitario Goiania GO.

**Keywords:** communication, sign language, hearing-impaired persons, relation between physician and patient, deafness

## Abstract

Non-verbal communication is important when caring for deaf patients, fostering excellence in health care. **Aim:** an analysis of the legal and sociocultural aspects of the relation between deaf patients and physicians. **Methods:** Computerized databases for the period between 1996 and 2006 were used for collecting data; the keywords “patient”, “deaf person”, “communication” and “health” were used. A non-systematic search was made in scientific publications. **Results:** These studies were grouped into two categories: communication between deaf patients and physicians, who has to deal with communication barriers when caring for deaf patients, and the importance of non-verbal communication in healthcare. Deaf persons, their language, and their relation with physicians show the importance of sign language, endorsed by the Federal Law 10.436/02. **Conclusions:** When deaf patients and physicians meet, they need to overcome communication barriers that may hinder the necessary bond in healthcare and the care that is provided; this may also affect the diagnosis and treatment. It is clear that public institutions should create programs for training healthcare professionals in the appropriate care of deaf patients.

## INTRODUCTION

The year 2000 census of the Brazilian Geographical and Statistical Institute (IBGE) shows that there are 24.5 million physically impaired people in Brazil, which is about 14.5% of the country’s population. Of these, 16.7% are hearing impaired; there are, therefore, 5,735,099 (five million, seven hundred and thirty five thousand and ninety nine) deaf people in Brazil.^1^

Interpersonal communication problems may be found in every health system; these issues become more significant when involving language and cultural barriers. The community of deaf persons, which uses sign language for communicating, faces significant hurdles when trying to access health services.^2^

The word “communicate” come from the Latin communicare, which means to “put in common.” It assumes a measure of understanding between involved parties. Two issues, then, become pertinent: can medical doctors understand the non-verbal expression of their deaf patients? And can deaf individuals understand the information their doctors convey?

The information that patients receive support their relationship with medical doctors, and may reduce their feelings of isolation, as well as increasing their satisfaction with and adherence to treatment. Medical doctor have a duty to communicate health-related issues (diagnosis and therapy), and patients have the right to receive such information.^3^, ^4^

The relationship between medical doctors and normal hearing patients is usually based on verbal codes, which is not the case of deaf patients. Thus, another channel is required for communication; usually, this is sign languge.^5^ This language, however, is almost never understood by those providing health care. Adequate treatment, in technical and human aspects, requires an analysis of how discursive formations are built, of the bonds that are made, and of relations that are formed within this scenario.

In 2003, the World Health Organization introduced a new inclusive model for assessing disabilities in general, the International Classification of Functioning, Disability and Health (ICF). Until this point, people with disabilities were assessed based on International Classification of Diseases (ICD) parameters. The ICD evaluates patients from the perspective of disease, while the ICF assesses persons from a functional standpoint. Brazil signed an agreement with other countries to officially adopt this new classification in 2004.^6^

As Rizzo^6^ points out, adoption of the ICF does not eliminate the need for the ICD, “Information about the diagnosis and about function enable a wider view of people’s realities, which facilitates decisions about possible interventions.” The analogy is worth repeating, “It is as if the ICD was a picture and the ICF was a movie.”

Admitting the complexity of the relation between physicians and deaf patients, its legal basis, and the identity and the cultural factors that characterize the community of hearing-impaired people is the basis for understanding such factors and providing essential service quality for this population.

## PURPOSE

The purpose of this paper was to review the publications of the Health Virtual Library (Biblioteca Virtual em Saude) that have dealt with legal, social and cultural aspects of the relation between deaf patients and physicians.

## METHOD

This review was undertaken in June 2006 on the Health Virtual Library (Biblioteca Virtual em Saude), using the LILACS (Latin American and Caribbean Health Science Literature) and MEDLINE (international medical literature) databases. The study period extended from 1996 to 2006; the key words were “patient,” “deaf,” “health,” and “communication.” A non-systematic search was also made in science journals about the relation between physicians and patients.

## RESULTS AND DISCUSSION

### Communication between deaf patients and listening physicians

Deaf patients use the health system differently from normal hearing patients; they report difficulties expressed as fear, mistrust and frustration. As a results of this they seek medical help less frequently.^7^ This becomes clear in the statement of a patient: “I was afraid I was not going to be understood; I had to wait five days until my mother arrived to go there with me.”

There is an undeniable need for improved communication between physicians and deaf patients; communication with deaf patients, however, remains neglected in health systems.^8^ Thus, non-verbal language is a form of communication that needs to be understood and validated in health services. Even not knowing sign language, it is essential to be able to interpret its suprasegmental aspects, such as gestures, and facial and body expressions.^9^ Another patient talked about this as follows: “It would be nice if healthcare workers understood sign language. Disease often cannot wait until an interpreter is found; things can get worse. Its what happened with me.”

Generally, physicians are not sufficiently prepared to care for deaf patients; academic curricula do not include the necessary abilities to meet the needs of this population group.^10^ Undoubtedly, effective communication with deaf patients is important in health care; inadequate communication may lead to wrong diagnoses and misguided therapy.^11^

As physicians become more specialized, patients are seen in a more fragmented manner, giving rise to a gap between the medicine of diseases and the medicine of patients, who receive care from many healthcare workers without connecting to any one of them. Human beings are not the sum of their parts, but rather an inseparable biological, psychic and social unit.^12^

Written language could be a way of overcoming communication difficulties with hearing impaired or deaf people that communicate orally; it is, however, inadequate for people that became deaf before acquiring oral language, and who learnt sign language as their first language.^10^ Portuguese is a second language for such people; as any foreign language, it may be hard to learn.^9^ This is clear in a comment that a patient made in sign language: “Doctors write down the time when you have to take medication, that is easy. What is hard is to understand the explanations about the disease, what is the medication for.”

Competency in establishing efficient communication and respecting cultural differences should include non-verbal communication and the ability to perceive and decode messages sent by patients.^4^ One of the most important factors that affects the quality and adequacy of care provided to deaf patients is the lack of awareness by healthcare workers about deaf people; added to this is a lack of ability to communicate non-verbally.^13^

The experience of deaf patients seeking health care shows that the quality of health care is improved when they have interpreters, or when physicians know sign language or at least attempt to improve communication by using pictures, drawings and non-verbal expressions.^7^

### Deaf patients, their language and the relation with physicians

Deafness is characterized by variably decreased perception of sound, which makes it difficult to acquire oral language naturally. The deaf community uses sign language as their first communication method, which is one of the factors that generates a feeling of belonging to a culture, although not all deaf people think of themselves as members of a community with unique features, language and social norms. The important fact is that deafness is different from other disabilities, not in itself, but by the difficulty in communicating with others.^14^

Sign language developed in all continents to overcome these barriers. The LIBRAS (Brazilian Sign Language) has been legally recognized in Brazil as a communication and expression method for the deaf community (Federal Law n^º^ 10.436/02). The decree n^º^ 5.626 was published in the “Diario Oficial da Uniao” on 22 December 2005 to regulate the matter. Article 25, Chapter VII, states that:

“As of one year after this Decree is published, the Sistema Unico de Saude (Unified Health System - SUS) and companies that own concessions or permits by public health care services, with the aim of fully including deaf or hearing impaired people in all aspects of social life, should assure full health care, including all levels of complexity and medical specialties, as follows:

I preventive measures and actions for developing auditory health programs;
II specialized medical care and treatment according to the specific features of each case;
IX care for deaf or hearing impaired people in the SUS service network and companies that own concessions or permits for executing health care public services by healthcare workers trained in the use of LIBRAS and its translation/interpretation; and
X support for training professionals in the SUS service network in the use of LIBRAS and its translation/interpretation.”^15^

The Ministry of Health has written a manual “Persons With Disabilities And The Unified Health System” for physicians, nurses and other healthcare workers, with the wider goal of including people with disabilities in the social context. The following sentence is part of this manual: “Comprehensive health care for people with disabilities assumes specific care for their condition, namely specific services for dealing with disabilities, as well as diseases that are common to all citizens.”^16^

Approval of Federal Law n^º^ 10.436/02 resulted from the efforts made by the Brazilian deaf community that uses sign language. Public institutions are responsible for putting in place training programs in the care and treatment of deaf patients for healthcare workers.^17^

Sign language is not a choice; it is the language of the deaf community. Access to information in this language is not well-defined; there are barriers that make it difficult for patients to understands their diseases and the decisions made for their health.^18^ A survey in the USA has shown that deaf patients prefer care by physicians that know sign language or that are deaf themselves.^7^

Deaf people communicate in a spatial-visual language with specific cultural features, and may have significant gaps in health knowledge.^19^ As an example of this difficulty, we have the following statement by a deaf patient: “Communication with healthcare workers is difficult, they need to understand deaf people better; just a recipe is not enough.”

## CONCLUSION

The review showed that deaf patients and physicians face communication barriers that compromise the development of the bonds that are required in health care, which may negatively affect the diagnosis and treatment.

First of all, it is necessary to learn about the specific features of deaf identity and culture in order to facilitate communication abilities and the relationship between deaf patients and physicians.

According to the LIBRAS Law 10.436/02, the rights of deaf people have to be assured by providing adequate training for healthcare workers to care for this segment of the population.

Understanding the issues around healthcare for deaf people facilitates the interaction between patients and physicians, which may reduce the discomfort in the clinical setting.

Studies are needed to understand health care for deaf people in greater detail, since there is relatively little published material in the literature about this theme.
